# Impaired memory-guided attention in asymptomatic APOE4 carriers

**DOI:** 10.1038/s41598-019-44471-1

**Published:** 2019-05-31

**Authors:** Jacqueline Zimmermann, Claude Alain, Chris Butler

**Affiliations:** 1 Department of Psychology, University of Toronto M5S 3G3 and Rotman Research Institute, Baycrest Centre, M6A 2E1 Toronto, Canada; 2 Nuffield Department of Clinical Neurosciences, John Radcliffe Hospital, University of Oxford, OX3 9DU Oxford, UK

**Keywords:** Attention, Long-term memory

## Abstract

Attention and memory may be impaired in individuals at-risk for Alzheimer’s disease (AD), though standard cognitive assessments typically study the two in parallel. In reality, attention and memory interact to facilitate information processing, and thus a more integrative approach is required. Here, we used a novel auditory paradigm to assess how long-term memory for auditory scenes facilitates detection of an auditory target in asymptomatic carriers of Apolipoprotein E4 (APOE4), the principle risk gene for late-onset AD. We tested 60 healthy middle-aged adults with varying doses of APOE4 - 20 APOE4 homozygotes (E4/E4), 20 heterozygotes (E3/E4) and 20 non-carriers (E3/E3) - to determine effect on memory-guided attention. While explicit memory was unaffected by genotype, APOE4 dose significantly impaired memory-guided attention. A relationship between explicit memory and memory-guided attention was observed in non-carriers, but this correlation was not significant in E3/E4 and E4/E4 carriers, suggesting that APOE4 carriers rely less on explicit memory to facilitate attention. Since memory-guided attention declined with age in APOE4 homozygotes, this impairment may reflect early disease rather than being a life-long trait. In sum, asymptomatic individuals at increased genetic risk of AD show an age-dependent decline in attention-memory interaction when memory alone is not impaired.

## Introduction

APOE, the gene encoding Apolipoprotein E, is the strongest marker of genetic susceptibility for late-onset Alzheimer’s disease (AD)^1^. The APOE4 allele is related to AD in a gene-dose dependent manner, with heterozygous E3/E4 carriers and homozygous E4/E4 carriers respectively having a 2–3 fold and 10–12 fold increased risk of disease^1^. APOE4 is implicated in the early accumulation of AD pathology in the brain, many years prior to the clinical onset of dementia^2^. However, evidence from meta-analyses suggests that the influence of APOE4 genotype upon cognitive function in healthy older adults is relatively small^3,4^. In middle-aged adults, when AD pathology may be at its very earliest stages, the data are equivocal^5,6^.

It is well known that accumulation of beta-amyloid in the hippocampus, which is an episodic memory hub^7,8^, occurs years before neurodegeneration and clinically detectable memory decline^2^. Increased beta-amyloid aggregation is also observed in early AD in the superior and posterior parietal cortex^2,9–11^, areas which are part of a network involved in orienting attention towards a stimulus or its memory representation^12–14^.

Episodic memory impairment, which is closely associated with hippocampal pathology^15^, is one of the first symptoms of AD and has been reported in healthy older APOE4 carriers using a variety of tasks and paradigms^16–18^. Long-term memory specifically is impaired for object locations in older E4/E4 carriers, whereas short-term memory for object locations is often intact or superior in this group^19^. Deficits in selective attention accompany memory impairment in early AD^20,21^, and have been related to beta-amyloid aggregation in the fronto-parietal attention network^22–24^.

In the majority of empirical research to date, and particularly in AD research, attention and memory have been tested separately. Yet real-world cognition involves constant interaction between memory and attention. Research using healthy individuals shows that attentional allocation is guided by prior experience, including episodic memory. For instance, in contextual cueing studies, participants take advantage of familiar contexts to guide search for a visual target^14,25–28^. The posterior parietal cortex has direct anatomical connections to the hippocampus and coherent spontaneous activity is observed between these regions^29^. Memory-guided attention, where selection of targets is aided by long-term memory (LTM) for relevant contexts, appears to recruit a hippocampal-parietal network that modulates top-down attention and interacts with the fronto-parietal attentional network involved in selective attention tasks^23,30^. Since early AD pathology targets both the hippocampus and superior and posterior parietal cortex, important hubs for memory and attention respectively, it is plausible that tasks that require an interaction of memory and attention will capture early AD-related deficits better than simple memory and attention tasks alone.

In vision research, there is a handful of studies showing that contextual cueing is impaired in individuals with mild cognitive impairment^31^ and even in healthy older APOE4 carriers^32^. Non-carriers show greater benefit compared with APOE4 carriers in detecting a target within arrays when the spatial arrangement of distracters is repeated from previous trials, indicating that memory-guided attention may function differently in at-risk individuals that otherwise perform normally on standard cognitive tests. However, in the visual-cue research, significant differences in memory-guided attention between carriers and non-carriers were observed in an elderly cohort (*M*_carriers_ = 78.5 years, *M*_noncarriers_ = 74.5 years^32^). This research was also limited because deficits observed in elderly individuals carry less predictive value since the age group of interest for early detection of AD is likely to be late middle-age. Also, the results may have been confounded by the presence of non-AD neuropathology more prevalent in older age. Moreover, recent work testing visuospatial attention in middle-aged adults, between the ages of 45 and 55, has found no impairment at this age in APOE4 carriers^33^.

With the focus in memory and attention research thus far being on visual paradigms, auditory processing has been largely neglected. However, memory for non-verbal auditory information is known to be poorer than memory for visual or tactile information^34^. Recent evidence shows that higher-order auditory processing deficits become evident in AD years before clinical symptoms arise, and that AD pathology affects brain regions implicated in processing auditory objects^35^. Notably, early AD patients show longitudinal decline in the ability to attend selectively to auditory information, and to recognize/interpret sounds, deficits that reflect impaired central auditory processing^36^ and auditory scene analysis^37^.

In simple listening conditions, individuals rely primarily on bottom-up factors such as attentional salience to extract intelligible signals from an auditory stream. As listening conditions become more demanding, individuals depend on working memory and LTM to fill in missing information^38,39^. This interaction of auditory memory and attention is highly relevant to speech processing and orientation within familiar auditory environments. Here, in a novel auditory paradigm, spatially informative audio-clips were used as LTM cues to facilitate detection of a faint and lateralized embedded auditory target. We analyzed performance of middle-aged asymptomatic APOE4 homozygotes (E4/E4), APOE4 heterozygotes (E3/E4), and non-carriers (E3/E3), and showed that APOE4 gene impaired listeners’ ability to use LTM representations to steer auditory attention. Though the gene-dose effect was not significant, the data showed a trend towards auditory memory-guided impairment by gene-dose. The age of our sample (40–61 years of age) allowed us to examine changes in performance over two decades before expected age of onset of late-onset AD, for which APOE4 is a risk gene^1^.

## Materials and Methods

### Paradigm overview

In the initial learning phase, participants learned to associate binaural auditory scenes (i.e., audio-clips) with the location of an embedded difficult-to-detect auditory target. While half the audio-clips contained a target, allowing a target-context association to be created in memory (memory condition), the other half of audio-clips were not paired with an auditory target (neutral condition), thus serving as a control condition. After a one hour delay, participants were presented with the same audio-clips, but now with all of them having a lateralized target tone, or an infrequent catch tone. While memory trials were associated with a specific spatial memory for the location of the target acquired during the learning task, the neutral trials were equally familiar from the learning task, but were not associated with a specific contextual spatial memory for the target. In a prior study, we showed that participants were more accurate and faster to detect the target on memory cue trials where the audio-clip and the target were combined during the learning phase, compared to neutral cue trials where no memory association existed and thus attention was divided between the left and right auditory fields^40^. The facilitating effect of the memory cue reflects memory-guided attention.

### Participants

The final sample consisted of 60 healthy participants between 40 and 61 years of age recruited through the Oxford Biobank, comprised three groups with varying APOE4 gene doses: E4/E4 (*N* = 20), E4/E3 (*N* = 20), and E3/E3 (*N* = 20). One participant who had scored below the inclusion cut-off for screening with the Addenbrooke’s Cognitive Examination version 3 (ACE-III; cut-off <86/100) was excluded from the sample^41^. The mean age of the sample (51 years) was well below the mean age of symptom onset in APOE4 homozygotes (68 years) and heterozygotes (76 years)^42^. APOE2 carriers were not tested due to the relative infrequency of this allele^1^, and also to preclude potential confounding effects since the APOE2 genotype has been shown to be protective against AD in some studies^43,44^.

Participants selected from each genotype group were chosen randomly from the Oxford Biobank participant pool based on the age criteria set by the experimenters as well as the requirement for normal hearing and no history of major physical or psychological illness. Letters were mailed to all participants that fit the recruitment criteria (E3/E3 *N* = 2621; E3/E4: *N* = 908; E4/E4 *N* = 107) by an independent administrator at the Oxford Biobank who was otherwise uninvolved in the project. Twenty interested participants from each genotype group were then randomly selected and contacted to participate in the experiment. Genetic results were not divulged to participants at any stage, nor to the experimenter before data collection was completed.

All participants provided informed consent prior to taking part in the study in accordance with the Declaration of Helsinki. The study was approved by the South Central Oxford C Research Ethics Committee (08/H0606/133). All collected data were anonymised and stored securely in accordance with the Data Protection Act 1998.

It should be noted that the Oxford Biobank participant sample used in this experiment showed a higher level of education than that of the general UK population (*M* = 9.4 years; http://www.nationmaster.com/country-info/stats/Education/), even though education was matched across groups. However, the majority of research suggests that the effects of APOE4 and education on development of dementia are independent^45,46^.

All participants were right-handed, fluent in English, had no history of psychiatric, neurological, or other major illness and had normal or corrected-to-normal vision and normal hearing. Hearing was assessed using pure tone thresholds for octave frequencies ranging from 250 to 8000 Hz, with the criterion for normal hearing being thresholds lower than or equal to 25 decibels of Sound Pressure Level (dB SPL) and less than or equal to a 15 dB SPL difference between the two ears at each octave frequency.

Groups were matched for age (*F*(2,57) = 0.576, *p* = 0.57), gender (X^2^ (2, *N* = 60) = 1.01, *p* = 0.61), years of education (*F*(2,57) = 1.59, *p* = 0.21), as well as cognitive ability as per scores on the ACE-III (*F*(2,57) = 0.24, *p* = 0.79). No differences in parental history of dementia (*F*(2,57) = 0.26, *p* = 0.78), maternal grandparents’ history of dementia (*F*(2,56) = 0.73, *p* = 0.49) or paternal grandparents’ history of dementia (*F*(2,55) = 0.54, *p* = 0.58) were observed. One participant was excluded in the analysis of maternal grandparents’ history of dementia, and two participants were excluded in the analysis of paternal grandparents’ history of dementia, because the information was not known. Parental history, maternal grandparental history, and paternal grandparental history were calculated as a score between 0 and 2 (no parent with dementia = 0, one parent with dementia = 1, two parents with dementia = 2). A total family history score was also calculated and matched across groups (*F*(2, 55) = 1.04, *p* = 0.36). This score comprised a sum of parental history score and both grandparental history scores, with affected parents given a weighting of 2 and grandparents given a weighting of 1, for a maximum score of 8. Demographic variables are presented in Table 1.

**Table 1:** Demographic characteristics of final sample, by group.

Genotype group	E3/E3	E3/E4	E4/E4
*N*	20	20	20
Age	50.3 (48.09−52.51)	51.8 (49.59−54.01)	51.7 (49.49−53.91)
Total years of education	14.80 (13.45−16.15)	16.30 (14.95−17.65)	16.25 (14.90−17.60)
Gender (M/F)	13/7	14/6	11/9
Parental History (0, 1 or 2 parents) (/2)	0.25 (0.02−0.48)	0.35 (0.21−0.58)	0.35 (0.21−0.58)
Paternal Grandparent History (0, 1, or 2 grandparents) (/2)	0.10 (−0.07−0.27)	0.21 (0.03−0.39)	0.21 (0.03−0.39)
Maternal Grandparent History (0, 1, or 2 grandparents) (/2)	0.15 (−0.04−0.34)	0.25 (0.06−0.44)	0.32 (0.12−0.51)
Total family history* (/8)	0.75 (0.20−1.30)	1.21 (0.65−1.77)	1.26 (0.70−1.83)
ACE-III Score (/100)	94.55 (92.81−96.29)	95.05 (93.31−96.79)	94.20 (92.46−95.94)

Group means are shown where applicable, with 95% confidence intervals in parentheses. No differences were found between groups on any demographic variable.

Note: Family history was assessed based on the number of parents/grandparents (0, 1 or both) reported to have had clinical memory problems. *Total family history was calculated by summing number of parents/grandparents reported to have had clinical memory problems, with affected parents given a weighting of 2, and affected grandparents given a weighting of 1 (maximum score is 8).

The Addenbrooke’s Cognitive Examination version 3 (ACE-III) was administered to screen for cognitive impairment (Hsieh *et al*.^41^). Scores below 86 indicate cognitive impairment.

### Cognitive assessment

The ACE-III was administered to screen for cognitive impairment^41^, since the aim of the current study was to assess the impact of APOE genotype independent of and prior to AD symptomology. The ACE-III is a relatively brief neuropsychological screening test consisting of attention, memory, language, fluency and visuo-spatial ability tests. It has been widely adopted in clinical settings to detect cognitive impairment associated with AD and other forms of dementia. Only participants who scored in the normal range (i.e., greater than the 86/100 cut-off score) were included in the study.

### Subjective memory assessment

Subjective memory was evaluated to identify subtle changes in self-perceived memory ability. In prior work, subjective memory concerns have been identified in asymptomatic familial AD carriers, and co-occur with hippocampus-dependent memory decline^47^. The presence of subjective memory concerns is also related to abnormal changes in beta-amyloid and tau biomarkers in APOE4 carriers^48–50^, and is considered by some researchers to be the earliest symptomatic manifestation of AD^51^.

Here, subjective memory was assessed using the Everyday Memory Questionnaire (EMQ) consisting of 18 questions relating to the perceived frequency of memory problems and forgetting in everyday situations^52^. Responses were given on a six point scale ranging from 0 = “not at all” to 5 = “more than once a day”, and the total EMQ score was calculated from 90 points (5 × 18), with higher scores indicating larger subjective memory concerns.

### Apoliprotein E genotyping

APOE genotyping was performed by the Oxford Biobank, using Applied Bio-systems TaqMan® SNP Genotyping Assay, C_3084793_20 and C_904973_10 corresponding to APOE SNPs rs429358 and rs7412, respectively, and run with ABI 7900HT Fast Real-Time PCR system. Diplotypes corresponding to APOE E3/E3, APOE E3/E4 and APOE E4/E4 were then identified.

### Stimuli, tasks and procedure

92 binaural audio-clips were retrieved from “http://www.freesounds.org/.” The clips were chosen to maintain considerable semantic relevance in order to increase the likelihood that an appropriate association could be formed and labelled in LTM. Semantic relevance was first evaluated based on subjective experience of the experimenter. At this first step, 104 audio-clips were selected by the experimenter, which had been used in a separate experiment. As a second step, in a separate pilot study, 12 audio-clips were eliminated which were judged to have “little to no semantic relevance” on a 5-step scale. Clips were cut to a length of 2500 ms, with a 100 ms rise and fall time, down-sampled to a standard sampling rate of 44100 Hz. All stimuli were presented through BOSE QuietComfort 25 over-ear noise cancelling headphones, at a listening volume of 60 dB SPL on average across stimuli, with some sounds peaking at about 80 dB SPL. Acoustic stimuli and visual cues were presented using Presentation software (version 13, Neurobehavioral Systems, Albany, CA).

The auditory target, which was embedded within the audio-clips, was a 500 Hz pure tone with 200 ms duration. A 200 ms high-pitched 2000 Hz catch tone was embedded within a minority of audio-clips. The target and catch tone had a 20 ms rise and fall time, were sampled at a 44100 Hz frequency, and were presented at a volume that was adjusted for each participant to allow 80% detectability.

Following audiometric screening, cognitive assessment, and subjective memory assessment, participants filled out a basic questionnaire to collect demographic characteristics including age, sex, years of education, and family history of memory impairment (Table 1). Next, they completed the main experimental task, which consisted of four parts.

1. **Determining individual signal-to-noise (SNR) thresholds**. We used a two alternative forced choice procedure to estimate each participant’s threshold in detecting a pure tone target. On each trial, participants were presented with the same audio-clip twice (each audio-clip was 500 ms in duration) separated by a 500 ms silent interval. The audio-clip was selected from a set of four audio-clips (i.e., bird chirping, sandpaper, people murmuring, industrial machine). None of the audio-clips used to estimate participants’ thresholds were used in the subsequent phases of the study. A pure tone target (500 Hz, 500 ms in duration, 50 ms rise/fall time) was embedded in one of the two audio-clip presentations. Participants were asked to indicate by pressing a button in which of the two audio-clip presentations the target was embedded. The 79% threshold of detectability was estimated using a three-down one-up algorithm^53^. At the beginning of the test, the target intensity was set at 60 dB SPL and decreased by a factor of 5 after three correct responses using the Attenuation function in Presentation (Neurobehavioral Systems). The intensity was increased by a factor of 5 after a mistake. The threshold was calculated by taking an average of the last 8 of 12 reversals^54^. The target intensity at the threshold level was then used in the subsequent phases of the experiment.
2. **Learning**. A total of 92 audio-clips, presented binaurally for 2500 ms, were divided into target-present (memory cue) and target-absent (neutral cue) trials (46 each). In the memory trials, a pure tone target was paired with the audio-clip, presented in the left (23 trials) or right (23 trials) ear at random. In the neutral trials, no target was presented. Each participant was presented with the same 92 audio-clips over six learning blocks (552 trials in total) to promote a strong association between audio-clips and location of the target when present. In addition, to strengthen the target-audio-clip associations, trials for which participants made an incorrect response were repeated immediately until a correct response was made within each block. The order of trials was random within each block, and trials for each condition were counterbalanced across participants and groups.
  Within memory trials, the target tone was played at each participant’s SNR threshold at 2000 ms after sound onset, and lasted for 200 ms. Participants were instructed to listen for the location of the target within each audio-clip, and pressed the left, right or down arrow key on a keyboard when the target was played from the left side, right side, or if no target was present, respectively. Participants were given 2000 ms to respond following the offset of the audio-clip, and subsequently received visual feedback for 500 ms to indicate hit, incorrect response (i.e., responding ‘no target’ when a target was present, or responding to target at the incorrect location), or no response, followed by a 1000 ms interval before the onset of the next trial. Participants were asked to respond as quickly and as accurately as possible.
3. **Memory-guided attention**. A one hour retention interval separated learning and the memory-guided attention test. During this time, participants filled in questionnaires (screening, ACE-III, EMQ) and completed a simple verbal memory task relevant to a separate study^55^. Following the retention interval, participants completed 92 trials in which they had to detect the pure tone target within a binaural audio-clip (80 trials) and withhold responses during a minority of catch trials (12 trials). The intensity of the pure tone target and catch tone was adjusted for each participant according to the 79% SNR threshold established at the beginning of the study^40^.
  Figure 1 provides a trial overview. On each trial, the same audio-clip, selected from the learning phase, was presented twice. The first presentation (S1) served as a cue to orient attention and did not include the target nor the catch tone. The second presentation (S2) occurred 1000 ms after S1 offset and included the pure tone target or the catch tone. For memory trials, the target (46 memory target trials) or catch tone (6 memory catch trials) was always presented at the previously learned location. For neutral trials, which did not include a pure tone target during the learning phase, a target (46 neutral target trials) or catch tone (6 neutral catch trials) was presented to either the left or right ear at random. Participants were instructed to press the left or right keyboard arrow button as quickly and as accurately as possible when they heard the pure tone target, and withhold responses on catch trials. A 2500 ms time window was given for responses starting from the onset of the target, and 1000 ms preceded the onset of the next trial. The order of trials was random within each block, and trials for each condition were counterbalanced across participants and groups.

**Figure 1:**
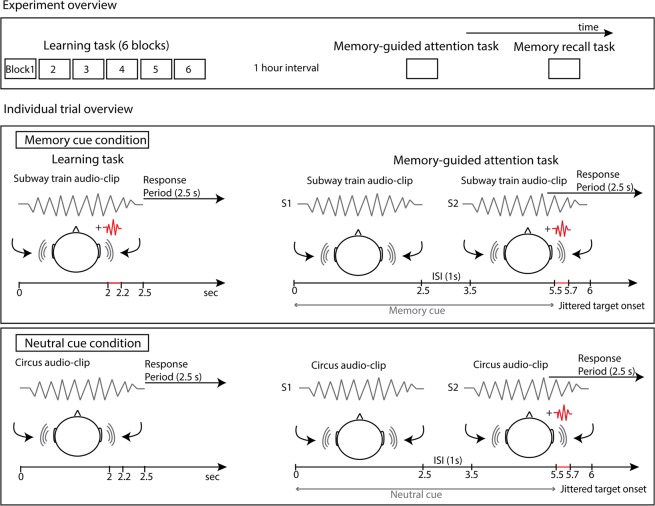
On top, an overview of the three main experimental tasks is shown: six block learning task (purpose: to create target-context associations), memory-guided attention task (purpose: to examine whether memory formed in the learning task guides attention) and explicit memory task (purpose: to determine whether and for which audio-clips the target location was consciously accessible). Below is an overview of one trial for each experimental condition, for the learning task and memory-guided attention task. In the memory cue condition, participants learned the location of the target, located in left or right hemispace, and the target was then presented at the learned location in the memory-guided attention task. RT and accuracy to detect the target was compared to neutral cue condition trials, where participants did not learn the location of a target (i.e., no target-context association formed in the learning task), and the target was then presented at a novel location in the memory-guided attention task. Note: S1 and S2 in the memory-guided attention task represent the first and second repetition of the audio-clip, separated by a 1000 ms ISI. Only S2 contained an embedded pure tone target, 2000 ms after S2 onset.
  The target was inserted towards the end of S2 (jittered between 1900–2100 ms after the audio-clip onset, 50 ms steps, rectangular distribution). A lengthy exposure to cues was intended to increase the informativeness of the memory cue and allow participants time to activate auditory memory for target location – context associations.
4. **Explicit memory**. A cued recall task was administered immediately after the memory-guided attention task in order to determine whether participants formed explicit associations between audio-clips and target location. Participants were presented with all 92 audio-clips, but without targets. For each audio-clip, participants indicated with a key-press response whether the target had been presented to the left or right ear or whether no target had been present in the learning phase. Participants were allowed as much time as needed to make their response. Subsequently, confidence in each response was rated using a 4-step scale key-press response, coding “I don’t know” responses as 0, “not very confident” responses as 1, “fairly confident” responses as 2, and “very confident” responses as 3.

### Experimental design and statistical analyses

The three APOE genotype groups were compared in a between-subjects design to identify differences in learning, explicit memory, memory-guided attention, and the relationship between explicit memory and memory-guided attention. Differences in explicit memory and memory-guided attention across groups were also analyzed across age.

#### Rate of auditory associative learning

A 3 × 6 mixed ANOVA was used to compare the rate of associative learning of audio-clip and target pairings across groups, with APOE4 genotype Group (E3/E3, E3/E4, E4/E4) as a between-subject variable, and Learning block (Block 1–6) as a within-subject variable. Response time (RT) and accuracy were input as dependent variables. RT was calculated from the time of target onset, and accuracy was calculated as a percentage of correct responses of all possible responses (incorrect plus too slow). Incorrect trials, trials where RT was faster than 100 ms or slower than 2500 ms (i.e., too slow to respond trials), as well as outliers (RT > 2.5 *SD* from the block mean) were excluded from the RT analysis. A between-subjects ANOVA was then used to confirm that all Groups reached the same level of performance by the last learning block.

#### Explicit memory

Accuracy during the explicit memory test and confidence ratings were compared across groups using a mixed ANOVA with APOE genotype Group (E3/E3, E3/E4, E4/E4), Explicit memory (correct, incorrect), and Confidence in responses (1 = not very confidence, 2 = fairly confident, 3 = very confident) as factors. Any “I don’t know” (0) responses were combined with incorrect responses in this analysis. In each group, regression analyses were performed to assess the effect of Age on accuracy. Because the primary aim was to determine whether Group is a moderating variable in the relationship between age and explicit memory, a multiple regression was also performed with Age and Group as regressors and explicit memory accuracy as the dependent variable. The Age × Group interaction term represented the moderating effect (i.e., whether the effect of age changed across groups).

#### Memory-guided attention

A 2 × 3 mixed ANOVA was used to assess the effects of APOE genotype on memory-guided attention (RT and accuracy), with Memory cue (memory cue vs neutral cue trials) as a within-subject variable, and APOE genotype Group (E3/E3, E3/E4, E4/E4) as a between-subject variable. Performance on the last trial of the learning task was input as a covariate in the memory-guided attention analysis to control for any differences in initial acquisition. In addition to fast response trials (<100 ms) and outliers (RT > 2.5 *SD* from the mean), error and catch trials were also excluded from the main analysis.

Regressions were set up to identify the effect of age on memory-guided attention for each group. Because we wanted to determine whether group is a moderating variable in the relationship between age and memory-guided attention, a multiple regression was set up with Age and Group as regressors and memory-guided attention as the dependent variable. The latter was calculated as a normalized difference score in RT to detect the target within memory and neutral audio-clip cues (*M*_Neutral_ − *M*_Memory_/*M*_Neutral_ + *M*_Memory_). The Age × Group interaction term represented the moderating effect. It should be noted that we calculated normalized difference scores in RT as opposed to accuracy because we expected that the ability for memory representations to facilitate speed in younger non-carriers would be pronounced compared to accuracy gains^56^.

In addition, to examine the relationship between explicit memory and memory-guided attention, a regression coefficient was calculated for each group. Explicit memory accuracy was input as the regressor, and memory-guided attention was operationalized with the normalized RT difference score. To assess whether this relationship changed across groups, a linear regression was set up with Explicit memory and Group as regressors, and normalized RT difference score as the dependent variable. The interaction of Explicit memory and Group reflected changes in the relationship between explicit memory and memory-guided attention across groups. We confirmed that Explicit memory and Group were not collinear (Variance Inflation Factor = 1.01).

We performed two subsidiary analyses to confirm that the benefit for memory cue trials reflected a biasing of spatial attention rather than a general increased readiness to respond. In the first analysis, we assessed whether the memory-guided benefit remains on those trials where participants recalled the target location at the wrong hemispace (e.g., recalled left when the target had been learned on the right), that is, where explicit memory acted as an invalid cue. Within-subject pairwise contrasts were used to compare RT on explicitly remembered memory cue trials, neutral cue trials, and invalid memory cue trials. To note, only those participants that had a sufficient number of invalid cue trials (i.e., 8 or more memory cue trials that were incorrectly recalled) and valid memory cue trials (i.e., 8 or more memory cue trials that were correctly recalled) were included in this analysis. Eight trials from each condition were chosen as the cut off to allow enough power for statistical analysis while maintaining a sufficient sample size (*N* = 22). As a secondary confirmation that the data cannot be understood as a generic increase in attention, we performed a within-subjects ANOVA comparing ability to withhold responses to catch trials associated with a memory cue and neutral cue. If the effect was simply driven by a generic increase in readiness in memory cue trials, then performance on catch trials that had previously been associated with a target location would also be affected.

#### Subjective memory assessment

Total EMQ scores (/90) were compared across Groups with a between-subjects ANOVA. To determine the association between subjective memory concerns and explicit memory, a non-parametric Spearman correlation (rho) was calculated between explicit memory accuracy and EMQ scores for each group^47^. To determine the association between subjective memory concerns and confidence in explicit memory, rho was also calculated between EMQ scores and total confidence scores for each group. Total confidence scores were quantified as the sum of confidence scores across all recall trials for each participant, with confidence scores for each trial being a number between 0 and 3.

## Results

### Rate of auditory associative learning

**Figure 2:**
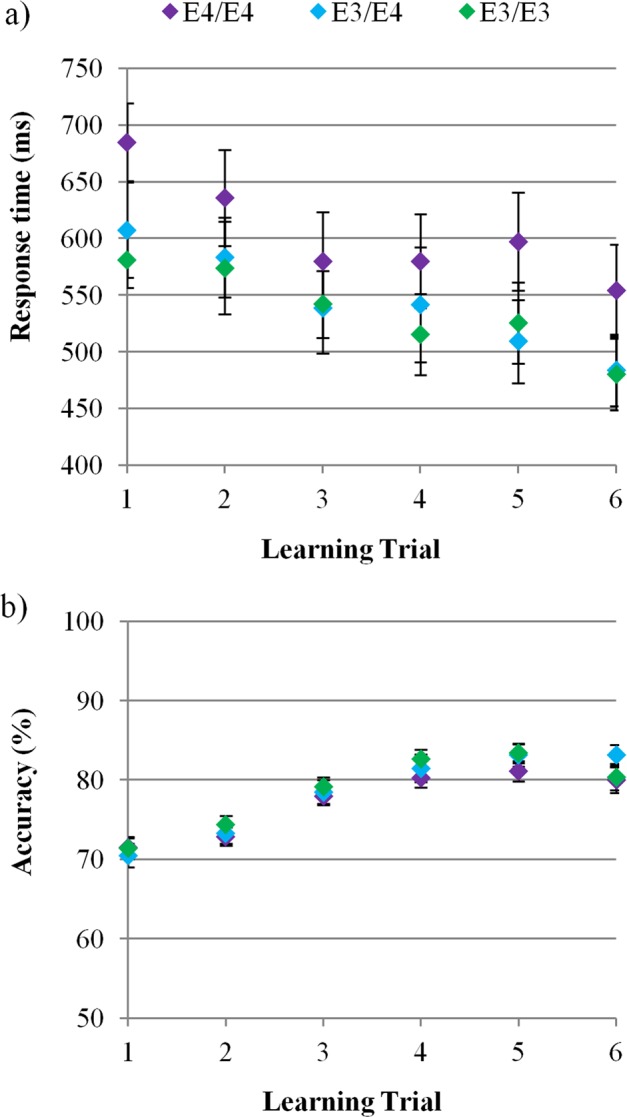
Learning of target-context associations over trials was unaffected by APOE genotype. (**a**) Participants became faster to detect targets over learning trials (group mean response time as a function of learning block: *F*(5,285) = 10.63, *p* < 0.001), but this change did not differ significantly across groups (Group × Trial: *F*(10,285) = 0.38, *p* = 0.96). (**b**) Participants became more accurate to detect targets over learning trials (group mean accuracy as a function of learning block: *F*(5,285) = 61.91, *p* < 0.001), but this change did not differ significantly across groups (Group × Trial: *F*(10,285) = 0.82, *p* = 0.61). Data for target-present trials only are shown. Error bars represent SEM; *N* = 20 per group.

Participants became faster (*F*(5,285) = 10.63, *p* < 0.001) and more accurate (*F*(5,285) = 61.91, *p* < 0.001) in detecting targets over learning trials. Importantly, learning did not differ significantly across groups (Group × Trial (RT): *F*(10,285) = 0.38, *p* = 0.96, Group × Trial (Accuracy): *F*(10,285) = 0.82, *p* = 0.61) (Fig. 2). There was no main effect of group for RT (*F*(2,57) = 1.42, *p* = 0.25) or accuracy (*F*(2,57) = 0.22, *p* = 0.80) during learning, or for performance on the last learning trial (RT: *F*(2,57) = 1.44, *p* = 0.25, Accuracy: *F*(2,57) = 0.87, *p* = 0.43). Hence, any subsequent group differences in performance could not be accounted for by differences in acquisition of the association between audio-clip and target.

### Explicit memory

One hour recall was unaffected by genotype (*F*(2,57) = 0.33, *p* = 0.72). Participants’ confidence in responses was associated with explicit memory correctness (Confidence × Correctness: *F*(2,56) = 84.30, *p* < 0.0001), but there were no differences in response confidence across genotypes (Group × Confidence: *F*(4,114) = 0.56, *p* = 0.69), and also no differences in the relationship between correctness and confidence in responses across genotypes (Group × Confidence × Correctness: *F*(4,114) = 0.69, *p* = 0.60).

**Figure 3:**
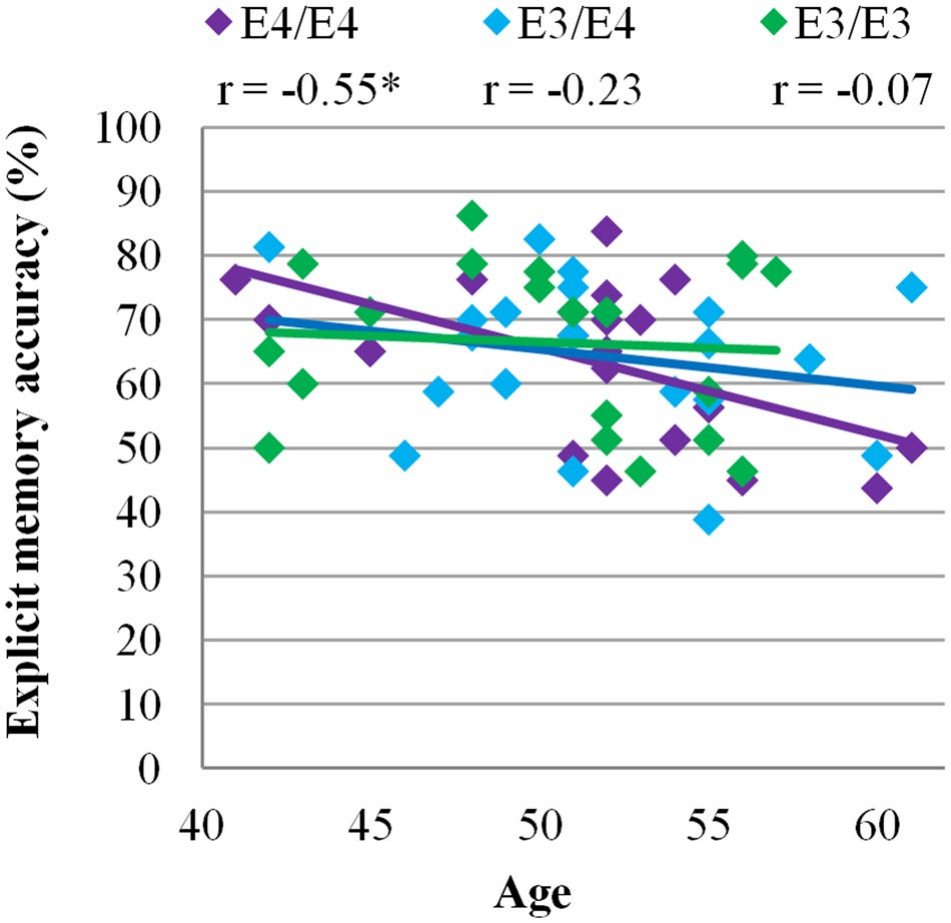
Cognitive performance across age in each APOE genotype group. Explicit memory declined significantly with age in E4/E4 carriers (*F*(1,19) = 7.60, *p* = 0.01, R = 0.55), but was not associated with age in E3/E4 (*F*(1,19) = 0.98, *p* = 0.34, R = 0.28) and E3/E3 groups (*F*(1,19) = 0.92, *p* = 0.77, R = 0.07). *N* = 20 per group.

Explicit memory declined significantly with age in E4/E4 carriers (*F*(1,19) = 7.60, *p* = 0.01, R = 0.55). However, this age effect was not observed in E3/E4 (*F*(1,19) = 0.98, *p* = 0.34, R = 0.28) and E3/E3 groups (*F*(1,19) = 0.92, *p* = 0.77, R = 0.07) (Fig. 3). The interaction of age and group was not significant (Age × Group: β = 0.51, *p* = 0.13).

### Auditory memory-guided attention

**Figure 4:**
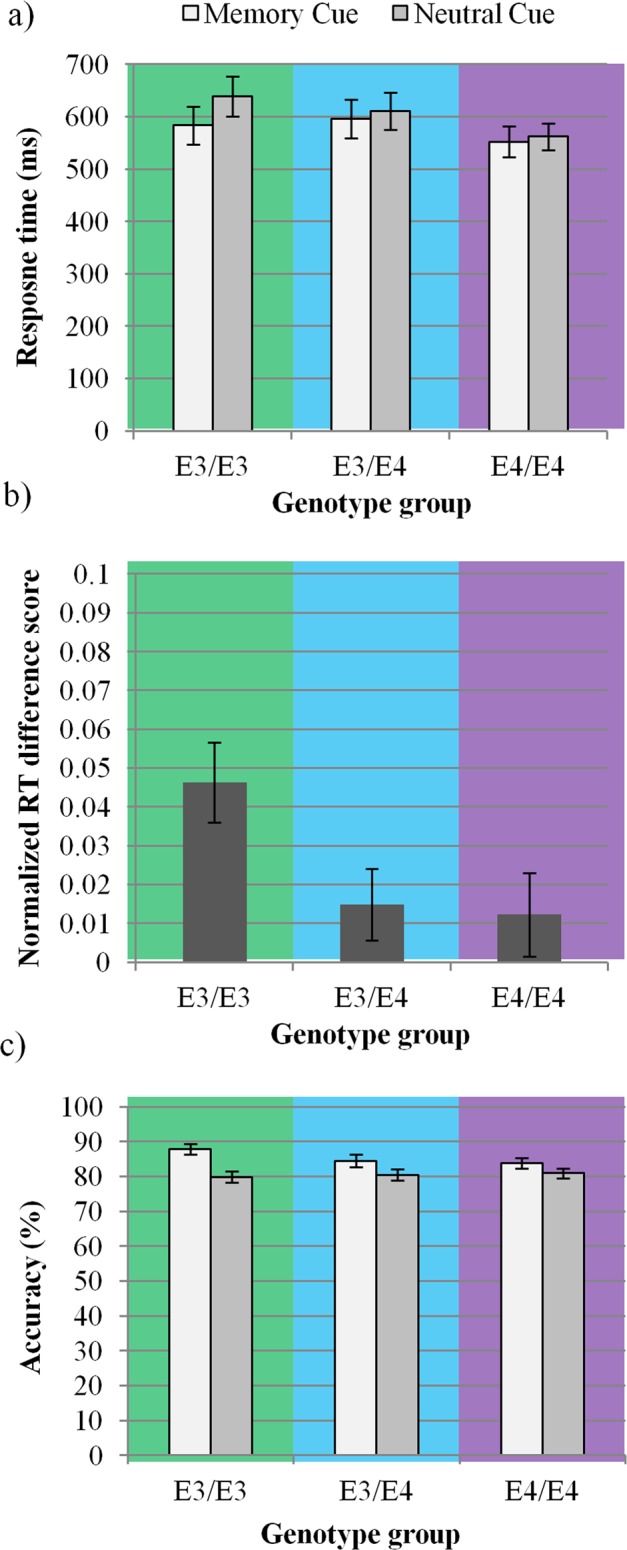
APOE4 dose significantly impaired memory-guided attention, in terms of cue effects on (**a**) generic response time (RT) (Memory cue × Group: *F*(2,57) = 4.21, *p* = 0.02), (**b**) normalized difference scores in RT (Group: *F*(1,56) = 5.70, *p* = 0.02) and (**c**) accuracy to detect the target (Memory cue × Group: *F*(2,57) = 6.00, *p* = 0.004). Note that in Fig. 4b, normalized RT difference score = *M*_Neutral_ − *M*_Memory_*/M*_Memory_ + *M*_Neutral_. The normalized difference score provides an understanding of the RT differences between conditions while controlling for group differences in overall speed. Cue-based gains in RT and accuracy were largest in E3/E3 genotype (*M*_Neutral-Memory_ = 55.69 ms, *M*_Memory-Neutral_ = 8.00%), compared to E3/E4 (*M*_Neutral-Memory_ = 15.10 ms, *M*_Memory-Neutral_ = 4.00%) and E4/E4 groups (*M*_Neutral-Memory_ = 9.96 ms, *M*_Memory-Neutral_ = 2.9%) which showed little to no memory-guided attention. Error bars represent SEM; *N* = 20 per group, 40 trials per condition.

There was a significant difference in memory-guided attention according to APOE genotype. Memory-based gains in response speed were largest in the E3/E3 group (*M*_Neutral-Memory_ = 55.69 ms), followed by the E3/E4 (*M*_Neutral-Memory_ = 15.10 ms) and the E4/E4 (*M*_Neutral-Memory_ = 9.96 ms) group (Memory cue × Group: *F*(2,56) = 4.21, *p* = 0.02; Fig. 4a). To determine whether the magnitude of memory-guided RT gains followed a gene-dose trend, we calculated linear contrasts for the effect of genotype Group on normalized memory-guided RT scores (*M*_Neutral_ − *M*_Memory_/*M*_Neutral_ + *M*_Memory_). We found that the magnitude of memory-guided attention was inversely proportionate to the dose of E4 alleles (i.e., E3/E3 > E3/E4 > E4/E4; *F*(1,56) = 5.70, *p* = 0.02; Fig. 4b). Pairwise comparisons confirmed that the E3/E3 group was significantly better at taking advantage of memory cues compared with the E3/E4 group (*p* = 0.03) and the E4/E4 group (*p* = 0.03). However, the E3/E4 group did not take advantage of cues significantly better than the E4/E4 group (*p* = 0.64).

We verified that there were no differences in variability in RTs for the two conditions across groups with an analysis of intra-individual standard deviation (ISD). The RT data used in the calculation of ISDs were cleaned beforehand to eliminate any responses faster than 100 ms and those outside 2.5 SD of the mean. We subsequently calculated ISD RT scores for each participant for both conditions separately across 1) all correct trials 2) the fastest 20% of those trials (fast ISD scores) 3) the slowest 20% of those trials (slow ISD scores). These scores were then transformed to T-scores to facilitate interpretation. We wanted to investigate both fast and slow ISD scores since high ISD scores reflect performance that is inconsistent across trials, whereas low ISD scores indicate consistent response performance. A mixed model ANOVA was used to assess the interaction of Group and Memory Cue on 1) all correct trial ISDs, 2) fast ISD scores and 3) slow ISD scores. Group did not interact with Memory cue to determine ISDs for any of the three ISD analyses. The lack of interaction for isolated fast ISD scores, in particular, confirmed that low-attention trials (slow ISDs) were not masking an effect.

Differences in accuracy were also observed as a function of APOE genotype. The benefits of the memory cue on accuracy were largest in the E3/E3 group (*M*_Memory-Neutral_ = 8.00%), followed by the E3/E4 (*M*_Memory-Neutral_ = 4.00%) and the E4/E4 (*M*_Memory-Neutral_ = 2.9%) group (Memory cue × Group: *F*(2,56) = 6.00, *p* = 0.004; Fig. 4c). To determine whether the benefit of memory cue on accuracy followed a gene-dose trend, we calculated linear contrasts for the effect of genotype Group on normalized memory-guided accuracy scores (*M*_Memory_ − *M*_Neutral_*/M*_Memory_ + *M*_Neutral_). As with the RT data, magnitude of memory-guided detection accuracy was inversely proportionate to the number of E4 alleles (i.e., E3/E3 > E3/E4 > E4/E4; *F*(2,56) = 7.45, *p* = 0.007).

We found that participants were successful at withholding responses during catch trials (77.75% correctly withheld), despite their infrequency. There was a difference between groups in ability to withhold responses (*F*(2.57) = 4.80, *p* = 0.01), such that E4/E4 carriers incorrectly responded to catch trials (72.87% correctly withheld) more often than did E3/E4 (80.41% correctly withheld) and E3/E3 groups (79.98% correctly withheld). Error trial RTs did not differ across groups when all three were included in the analysis (*F*(2.57) = 1.78, *p* = 0.18), though trends in group means revealed that E4/E4 carriers responded more quickly on incorrect trials than E3/E4 and E3/E3 groups (Error trials RT: E4/E4 *M* = 506.41; E3/E4 *M* = 573.81; E3/E3 *M* = 569.03).

**Figure 5:**
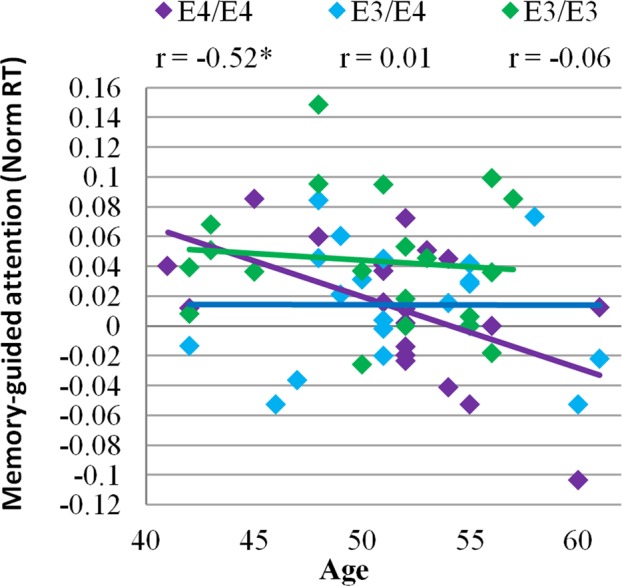
Cognitive performance across age in each APOE genotype group. Memory-guided gains in response time (RT) decreased with age in the E4/E4 group (*F*(1,19) = 6.31, *p* = 0.02, R = 0.52) but were not affected by age in E3/E4 (*F*(1,19) = 0, *p* = 0.97, R = 0.01) and E3/E3 (*F*(1,19) = 0.03, *p* = 0.82, R = 0.06) groups. Memory-guided gains in RT were calculated as a normalized difference score between RTs to detect targets preceded by neutral and informative memory cues (*M*_Neutral_ − *M*_Memory_*/M*_Memory_ + *M*_Neutral_). *N* = 20 per group.

Memory-guided attention effects on RT (*M*_Neutral_ − *M*_Memory_*/M*_Neutral_ + *M*_Memory_) decreased with age in the E4/E4 group (*F*(1,19) = 6.31, *p* = 0.02, R = 0.52), but were not affected by age in E3/E4 (*F*(1,19) = 0, *p* = 0.97, R = 0.01) and E3/E3 (*F*(1,19) = 0.03, *p* = 0.82, R = 0.06) groups (Fig. 5). Group was a moderating variable in the relationship between age and memory-guided attention (Age × Group: β = 0.65, *p* = 0.05).

**Figure 6:**
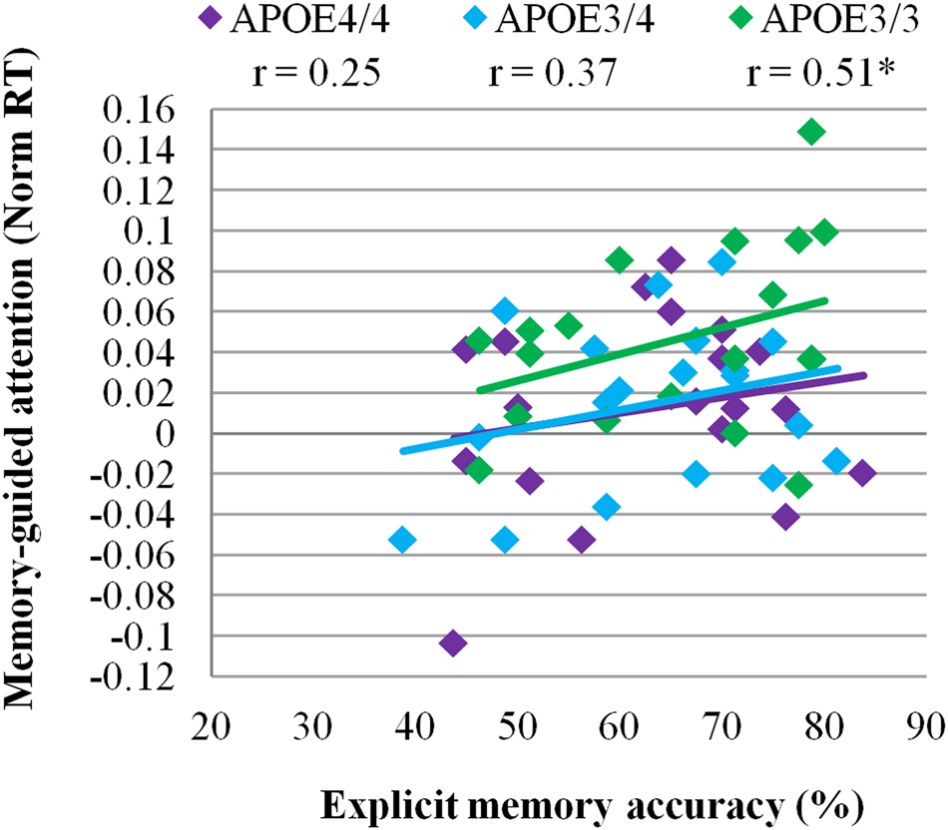
A relationship between memory and memory-guided attention was present only in E3/E3 group (*F*(1,19) = 5.95, *p* = 0.03, R = 0.51), but not in E3/E4 (*F*(1,19) = 2.67, *p* = 0.12, R = 0.37) and E4/E4 (*F*(1,19) = 0.12, *p* = 0.30, R = 0.25) groups. *N* = 20 per group.

A relationship between explicit memory and memory-guided attention was present in the E3/E3 group (*F*(1,19) = 5.95, *p* = 0.03, R = 0.51), but the relationship was not significant in E3/E4 (*F*(1,19) = 2.67, *p* = 0.12, R = 0.37) or E4/E4 (*F*(1,19) = 1.20, *p* = 0.30, R = 0.25) groups (Fig. 6). This relationship did not differ significantly between groups (Explicit memory × Group: β = 0.18, *p* = 0.78). A subsidiary analysis was performed to ascertain that the relationship between explicit memory and memory-guided attention remained significant in the E3/E3 group even after an outlying participant (Norm RT = 0.15, Mean recall = 78%) was excluded (see Fig. 6; *F*(1,18) = 4.64, *p* = 0.047, R = 0.47). In addition, when an outlying participant was excluded from the E4/E4 group (Norm RT = −0.10, Mean recall = 43%) the correlation between recall and memory-guided attention was further reduced (*F*(1,19) = 0.03, *p* = 0.86, R = 0.04).

Two subsidiary analyses of the whole sample data confirmed that the benefit elicited by memory cue trials reflected a biasing of spatial attention rather than a generic increase in readiness to respond. First, we found that there was a significant slowing on our surrogate ‘invalid’ trials, where participants had recalled the target’s location at the wrong hemispace, compared to explicitly remembered memory cue trials (Explicitly remembered memory cue trials vs Invalid trials: *F*(1,21) = 4.90, *p* = 0.038; Invalid trials vs Neutral cue trials: *F*(1,21) = 0.83, *p* = 0.372). Second, we found that participants’ ability to withhold responses to catch trials did not differ in the memory and neutral cue condition (*F*(1,59) = 1.04, *p* = 0.311).

### Subjective memory assessment

No differences in EMQ scores were observed between genotype groups (*F*(2,57) = 1.30, *p* = 0.28). There was no association between EMQ scores and explicit memory (E3/E3: rho = −0.24, *p* = 0.31; E3/E4: rho = 0.26, *p* = 0.27; E4/E4: rho = 0.26, *p* = 0.27), or between EMQ scores and confidence in explicit memory (E3/E3: rho = 0.10, *p* = 0.67; E3/E4: rho = 0.03, *p* = 0.90; E4/E4: rho = −0.08, *p* = 0.73) in any of the groups.

## Discussion

Using a novel auditory paradigm, we found that healthy, middle-aged APOE4 carriers showed reduced interaction between attention and memory for non-verbal environmental sounds compared with non-carriers. Primarily, we showed preserved learning and explicit memory in APOE4 carriers but impairment in memory-guided attention. Deficits in memory-guided attention were particularly striking given the age of our sample (40–61 years), the mean of which preceded the mean age of symptom onset in APOE4 carriers by over two decades^42^. Secondly, and of potential clinical importance, was the finding that memory-guided attention declined with age in only in APOE4 homozygotes, suggesting that the differences in cognitive performance may be disease-related rather than life-long.

Impaired memory-guided attention in APOE4 carriers was reflected in smaller gains in RT and accuracy to detect targets within informative contexts (memory cues) compared to uninformative contexts (neutral cues) (Fig. 4). In other words, APOE4 carriers had difficulty using informative contexts to facilitate attention. Moreover, mean RTs suggest that the magnitude of memory-guided attention was inversely proportional to APOE4 gene-dose (mean gains in memory-guided RTs: E3/E3 > E3/E4 > E4/E4). Direct comparisons between non-carriers and APOE4 heterozygotes as well as between APOE4 heterozygotes and APOE4 homozygotes confirm that non-carriers were indeed better at using memory to aid attention (i.e., higher normalized memory-guided attention score). However, APOE4 heterozygotes were not significantly worse than APOE4 homozygotes at using memory to aid attention (pairwise comparison statistical tests for gains in memory-guided RT: E3/E3 > E3/E4 = E4/E4). It may be the case that having one APOE4 allele significantly impairs performance, or that sample sizes were simply not large enough to capture the gene-dose trend.

Our findings suggest that assessing the interaction of attention and memory processes in at-risk individuals reveals impairment that is not evident when assessing associative recollection memory alone. Given that memory-guided attention declined with age in APOE4 homozygotes who are at the greatest risk for AD but was not significant in the other two groups (Fig. 5), it appears that memory-guided attention impairment may reflect disease-related pathology that increases with age. The deficits observed here in memory-guided attention were much earlier than those documented in a prior contextual cueing study in which APOE4 carriers over 70 year of age showed impairments in visual target detection in repeated spatial arrays^31^. Moreover, contextual cueing paradigms do not specifically assess the effect of LTM on attention, because learning and memory-guided attention cannot be parsed^14^. Here, target-context associations were acquired during a learning task, which was separate from the memory-guided attention task.

Our findings can be understood in the context of existing neuroimaging evidence, which reveals that both the hippocampus, a memory hub, and the superior and posterior parietal cortex, a key attention structure, are susceptible to accumulation of beta-amyloid which can begin up to 30 years before the onset of AD symptoms^9–11,57^. This process is accelerated by APOE4 genotype both in humans and animals^56,58^,^59^. A pattern of differential regional recruitment of superior parietal and hippocampal areas during attention and memory tasks can be seen as early as middle-age in APOE4 carriers^31,56,60–62^, and is often accompanied by decreased neural connectivity within and between these areas, suggesting impacts on memory/attention interactions^63–65^. Yet, in current practice, the majority of clinical AD assessments examine attention and memory in isolation. Our findings, together with the neuroimaging evidence discussed, suggest that probing memory-guided attention may be more effective at detecting early behavioural decline in AD.

We also found that APOE4 was associated with a reduced ability to withhold responses during catch trials, which may be related to inhibition of prepotent responses or a general failure to utilize context to aid accuracy. In either case, the catch trial findings support faulty top-down processing in APOE4. In the former case, contexts activated responses which APOE4 carriers could not inhibit. In the latter case, contexts were altogether ignored by APOE4 carriers. However, there were few catch trials and further investigation is needed for a conclusive interpretation. It should also be noted that when memory trials alone were analyzed, APOE4 carriers were not significantly slower than non-carriers, a finding that can be explained by an increase in baseline response speed in APOE4 carriers. That is, APOE4 carriers were generally speedier on neutral trials, and lacked inhibition, compared to non-carriers. The normalized measure of memory-guided attention takes this change in baseline speed into account, and therefore reflects true impairment in memory-guided attention in APOE4 carriers (see Fig. 4b). It seems likely that there could be other factors driving increased response speeds for neutral trials and reduced inhibition in APOE4 (see Fig. 4a), for instance an influence of antagonistic pleiotropy^66–69^. However, regardless of group differences in general speed, the differences between RTs for memory and neutral trials (i.e., normalized RT gains) reveal a clear impairment of memory-guided attention amongst APOE4 carriers (see Fig. 4b).

The gene-related performance declines observed here occurred independent of and despite absence of subjective memory concerns. It has previously been shown that subjective memory complaints may co-occur with subtle objective cognitive decline in individuals genetically at-risk for AD^47,70^. It is possible, therefore, that impaired memory-guided attention actually precedes both objective and subjective memory recollection deficits.

Though explicit memory did not differ significantly across APOE genotypes, we observed an effect of age on explicit memory in APOE4 homozygotes (Fig. 3). Just as with memory-guided attention, explicit memory performance of APOE4 homozygotes declined with age while performance of APOE4 heterozygotes and non-carriers remained stable with age. In studies of healthy aging, memory-related changes in brain function can be detected in middle-age when memory is assessed using spatial context memory tasks that require participants to make associative connections between stimuli^71,72^. However, impairments in behavioural recall in APOE4 carriers are typically observed much later in life^16–18^. A plausible explanation for the observed age effect in the absence of a general group effect is that APOE4 conveys a benefit in memory at younger ages and a deficit at older ages. This effect has been described by the antagonistic pleiotropy hypothesis^66^, which is supported in APOE4 by studies of episodic memory^67^ and visuospatial attention^68,69^. Moreover, neuroimaging reveals that older APOE4 carriers compensate for cognitive decline with additional neural activation in frontal, superior parietal and hippocampal areas, characteristic of the pattern of neural recruitment observed later in life^56^.

We also found a relationship between accessibility of memory cues (i.e., explicit memory for target-context associations) and memory-guided attention in non-carriers, which was absent in APOE4 heterozygotes and homozygotes (Fig. 6). This suggests that memory-guided attention in APOE4 carriers did not depend on the quality of the explicit memory trace, though a larger sample would be required to make a conclusive interpretation. Similar findings have been reported in one other study, which used informative visual scenes to cue attention in middle-aged APOE4 carriers^73^. The question arises whether APOE4 carriers rely on implicit memory to drive attention, compensating for effects of explicit memory. Since implicit memory was not directly measured in our study, it was not possible to answer this question.

There were important distinctions in the processing engaged by our participants compared to prior visual memory-guided attention work discussed. Our participants relied on activation of associations which were not as spatially sensitive (e.g., target at left ear in the cafe audio-clip) compared to the detailed contextual memory acquired in visual cueing tasks, where targets can appear anywhere within the visual scene. In contextual cueing tasks, context repetition facilitates visual search through reducing the number of distracters that must be processed during search^74^. In our paradigm, auditory cues required participants to activate simpler target-audio-clip associations, which could be characterized as target-item associations rather than target-context associations. This is because audio-clips, in contrast to visual scenes, do not contain numerous spatially distributed subcomponents, and are more likely to be processed as a unitary auditory object^75^.

Compared with the visual studies discussed here, the current auditory task appears particularly sensitive in detecting early cognitive changes. For example, Salvato *et al*.^73^ investigated an almost identical cohort (20 E3/E3, 20 E3/E4, 20 E4/E4; age range 40–60 years) recruited from the same participant database in Oxford, and used a similar experimental design to ours, but with photographs rather than audio-clips as cues. Notably, Salvato *et al*.^73^ failed to find any differences in memory-guided attention between APOE groups. It is possible that the difficulty in remembering a large set of audio-clip cues, compared to remembering a large set of visual scenes, contributed to the earlier decline in memory-guided attention^34^.

Moreover, though top-down visual and auditory attention are both sub-served by an attentional network centered in the parietal cortex^76–78^, there are notable modality differences within this network. In an endogenous attention task conducted by Smith *et al*.^79^ set up to examine modality differences, less than a third of the activated fronto-parietal network demonstrated modality-independent overlap. These differences are important because input to and from sensory regions is critical not only during preparation of attention, but also during activation of memory representations that are responsible for this preparation^14,79^. The visual cortex is affected in relatively later stages of AD, and shows little beta-amyloid accumulation in early stages. In contrast, numerous regions implicated in higher-order auditory processing, including inferior and mid-temporal gyri and temporoparietal gyri, are affected even in pre-clinical AD^80,81^. Listening in complex environments where participants must rely on working memory and LTM is impaired years before symptoms arise in some longitudinal studies^36,37,81^. This is the kind of processing employed by participants in the current study; LTM cues are used to facilitate perception of a difficult-to-detect target.

A significant advantage of the current study was the inclusion of three separate APOE4 gene-dose groups. Many prior studies have simply compared APOE4 carriers (homozygotes plus heterozygotes) with non-carriers, which can hide true cognitive impairment^82,83^. Though our study revealed an intriguing association between age and memory-guided attention in APOE4 homozygotes, it is important to note that the data were cross-sectional and the age range in the sample was relatively small. Future studies should examine memory-guided attention longitudinally in APOE4 carriers, as well as its relationship to AD biomarkers such as beta-amyloid accumulation, to ascertain the influence of pathology. Differences in neural substrates of memory-guided attention in APOE4 carriers could be examined using fMRI, to determine whether there is a decoupling of attentional and memory circuits^83^.

In conclusion, APOE4 genotype exerts influence on memory-guided attention in asymptomatic middle-aged individuals. Ability to use auditory LTM to guide target detection is impaired by APOE4, while initial learning and LTM recall are intact.

## Data Availability

The datasets generated during and/or analysed during the current study are available from the corresponding author on reasonable request.

## References

[1] Raber J, Huang YD, Ashford JW (2004). ApoE genotype accounts for the vast majority of AD risk and AD pathology. Neurobiology of Aging.

[2] Jack CR (2010). Hypothetical model of dynamic biomarkers of the Alzheimer’s pathological cascade. Lancet Neurology.

[3] Small BJ, Rosnick CB, Fratiglioni L, Backman L (2004). Apolipoprotein E and cognitive performance: A meta-analysis. Psychology and Aging.

[4] Wisdom NM, Callahan JL, Hawkins KA (2011). The effects of apolipoprotein E on non-impaired cognitive functioning: A meta-analysis. Neurobiology of Aging.

[5] Mayeux R, Small SA, Tang MX, Tycko B, Stern Y (2001). Memory performance in healthy elderly without Alzheimer’s disease: effects of time and apolipoprotein-E. Neurobiology of aging.

[6] Mecca AP (2018). Cortical beta-amyloid burden, gray matter, and memory in adults at varying APOE epsilon 4 risk for Alzheimer’s disease. Neurobiology of Aging.

[7] Moscovitch M, Cabeza R, Winocur G, Nadel L (2016). Episodic Memory and Beyond: The Hippocampus and Neocortex in Transformation. Annual Review of Psychology,.

[8] Rugg MD, Vilberg KL (2013). Brain networks underlying episodic memory retrieval. Current Opinion in Neurobiology.

[9] Drzezga A (2011). Neuronal dysfunction and disconnection of cortical hubs in non-demented subjects with elevated amyloid burden. Brain.

[10] Engler H (2006). Two-year follow-up of amyloid deposition in patients with Alzheimer’s disease. Brain.

[11] Mintun MA (2006). (11) PIB in a nondemented population - Potential antecedent marker of Alzheimer disease. Neurology.

[12] Astle DE, Summerfield J, Griffin I, Nobre AC (2012). Orienting attention to locations in mental representations. Attention Perception & Psychophysics.

[13] Nobre AC (2004). Orienting attention to locations in perceptual versus mental representations. Journal of Cognitive Neuroscience.

[14] Summerfield JJ, Rao A, Garside N, Nobre AC (2011). Biasing Perception by Spatial Long-Term Memory. Journal of Neuroscience.

[15] Mormino EC (2009). Episodic memory loss is related to hippocampal-mediated -amyloid deposition in elderly subjects. Brain.

[16] Adamson MM (2010). Apolipoprotein E epsilon 4 influences on episodic recall and brain structures in aging pilots. Neurobiology of aging.

[17] Bailey HR (2015). APOE epsilon 4 genotype predicts memory for everyday activities. Aging Neuropsychology and Cognition.

[18] De Blasi S (2009). APOE polymorphism affects episodic memory among non demented elderly subjects. Experimental gerontology.

[19] Zokaei N (2019). Dissociable effects of the apolipoprotein-E (APOE) gene on short- and long-term memories. Neurobiology of Aging.

[20] Bonney KR (2006). Inspection time in non-demented older adults with mild cognitive impairment. Neuropsychologia.

[21] Parasuraman R, Greenwood PM, Sunderland T (2002). The apolipoprotein E gene, attention, and brain function. Neuropsychology.

[22] Hahn K (2013). Selectively and progressively disrupted structural connectivity of functional brain networks in Alzheimer’s disease - Revealed by a novel framework to analyze edge distributions of networks detecting disruptions with strong statistical evidence. Neuroimage.

[23] Neufang S (2014). Predicting effective connectivity from resting-state networks in healthy elderly and patients with prodromal Alzheimer’s disease. Human Brain Mapping.

[24] Sorg C (2007). Selective changes of resting-state networks in individuals at risk for Alzheimer’s disease. Proceedings of the National Academy of Sciences of the United States of America.

[25] Chun MM, Jian YH (1998). Contextual cueing: Implicit learning and memory of visual context guides spatial attention. Cognitive psychology.

[26] Chun MM, Jiang YH (2003). Implicit, long-term spatial contextual memory. Journal of Experimental Psychology-Learning Memory and Cognition.

[27] Patai EZ, Doallo S, Nobre AC (2012). Long-term Memories Bias Sensitivity and Target Selection in Complex Scenes. Journal of cognitive neuroscience.

[28] Stokes MG, Atherton K, Patai EZ, Nobre AC (2012). Long-term memory prepares neural activity for perception. Proceedings of the National Academy of Sciences of the United States of America.

[29] Vincent JL (2006). Coherent spontaneous activity identifies a hippocampal-parietal memory network. Journal of Neurophysiology.

[30] Goldfarb EV, Chun MM, Phelps EA (2016). Memory-Guided Attention: Independent Contributions of the Hippocampus and Striatum. Neuron.

[31] Negash S (2015). Relationship of Contextual Cueing and Hippocampal Volume in Amnestic Mild Cognitive Impairment Patients and Cognitively Normal Older Adults. Journal of the International Neuropsychological Society.

[32] Negash S (2007). Effects of ApoE genotype and mild cognitive impairment on implicit learning. Neurobiology of aging.

[33] Lancaster C, Forster S, Tabet N, Rusted J (2017). Putting attention in the spotlight: The influence of APOE genotype on visual search in mid adulthood. Behavioural Brain Research.

[34] Bigelow James, Poremba Amy (2014). Achilles’ Ear? Inferior Human Short-Term and Recognition Memory in the Auditory Modality. PLoS ONE.

[35] Goll JC (2011). Auditory object cognition in dementia. Neuropsychologia.

[36] Gates GA, Anderson ML, McCurry SM, Feeney MP, Larson EB (2011). Central Auditory Dysfunction as a Harbinger of Alzheimer Dementia. Archives of Otolaryngology-Head & Neck Surgery.

[37] Golden HL (2016). Functional neuroanatomy of spatial sound processing in Alzheimer’s disease. Neurobiology of aging.

[38] Arlinger S, Lunner T, Lyxell B, Pichora-Fuller MK (2009). The emergence of Cognitive Hearing Science. Scandinavian Journal of Psychology.

[39] Lee SJ, Kim H (2016). Effect of Keyword Position on Sentence Recognition under Background Noise in Mild Cognitive Impairment. Communication Sciences and Disorders-Csd.

[40] Zimmermann JF, Moscovitch M, Alain C (2017). Long-Term Memory Biases Auditory Spatial Attention. Journal of Experimental Psychology-Learning Memory and Cognition.

[41] Hsieh S, Schubert S, Hoon C, Mioshi E, Hodges JR (2013). Validation of the Addenbrooke’s Cognitive Examination III in Frontotemporal Dementia and Alzheimer’s Disease. Dementia and geriatric cognitive disorders.

[42] Liu CC, Kanekiyo T, Xu H, Bu GJ (2013). Apolipoprotein E and Alzheimer disease: risk, mechanisms and therapy (vol 9, pg 106, 2013). Nature Reviews Neurology.

[43] Marques-Vidal P (2003). Obesity and alcohol modulate the effect of apolipoprotein e polymorphism on lipids and insulin. Obesity research.

[44] Shinohara M (2016). APOE2 Eases Cognitive Decline during Aging: Clinical and Preclinical Evaluations. Annals of Neurology.

[45] Anttila T (2002). Midlife income, occupation, APOE status, and dementia - A population-based study. Neurology.

[46] Ngandu T (2007). Education and dementia - What lies behind the association?. Neurology.

[47] Weston PSJ (2018). Accelerated long-term forgetting in presymptomatic autosomal dominant Alzheimer’s disease: a cross-sectional study. Lancet Neurology.

[48] Risacher SL (2015). APOE effect on Alzheimer’s disease biomarkers in older adults with significant memory concern. Alzheimers & Dementia.

[49] Zwan MD (2016). Subjective Memory Complaints in APOE epsilon 4 Carriers are Associated with High Amyloid-beta Burden. Journal of Alzheimers Disease.

[50] Zhang J (2018). Risk factors for amyloid positivity in older people reporting significant memory concern. Comprehensive Psychiatry.

[51] Rabin LA, Smart CM, Amariglio RE (2017). Subjective Cognitive Decline in Preclinical Alzheimer’s Disease. Annual Review of Clinical Psychology,.

[52] Sunderland A, Harris JE, Gleave J (1984). Memory Failures in Everyday Life Following Severe Head-Injury. Journal of Clinical Neuropsychology.

[53] Levitt H. (1971). Transformed Up‐Down Methods in Psychoacoustics. The Journal of the Acoustical Society of America.

[54] Ellis RJ, Sorqvist P, Zekveld AA, Ronnberg J (2017). Editorial: Cognitive Hearing Mechanisms of Language Understanding: Short- and Long-Term Perspectives. Frontiers in Psychology.

[55] Zimmermann JF, Butler CR (2018). Accelerated long-term forgetting in asymptomatic APOE epsilon 4 carriers. Lancet Neurology.

[56] Evans S (2014). Cognitive and neural signatures of the APOE E4 allele in mid-aged adults. Neurobiology of Aging.

[57] Buckner RL (2005). Molecular, structural, and functional characterization of Alzheimer’s disease: Evidence for a relationship between default activity, amyloid, and memory. Journal of Neuroscience.

[58] Zhao N, Liu CC, Qiao WH, Bu GJ (2018). Apolipoprotein E, Receptors, and Modulation of Alzheimer’s Disease. Biological Psychiatry.

[59] Youmans KL (2012). APOE4-specific Changes in A beta Accumulation in a New Transgenic Mouse Model of Alzheimer Disease. Journal of Biological Chemistry.

[60] Mormino EC (2012). A beta Deposition in Aging Is Associated with Increases in Brain Activation during Successful Memory Encoding. Cerebral Cortex.

[61] Nichols LM (2012). Interactive Effect of Apolipoprotein E Genotype and Age on Hippocampal Activation During Memory Processing in Healthy Adults. Archives of General Psychiatry.

[62] Scheller E (2017). APOE moderates compensatory recruitment of neuronal resources during working memory processing in healthy older adults. Neurobiology of Aging.

[63] Sheline YI (2010). APOE4 Allele Disrupts Resting State fMRI Connectivity in the Absence of Amyloid Plaques or Decreased CSF A beta 42. Journal of Neuroscience.

[64] Heise V (2014). Apolipoprotein E genotype, gender and age modulate connectivity of the hippocampus in healthy adults. Neuroimage.

[65] Habib M (2017). Functional neuroimaging findings in healthy middle-aged adults at risk of Alzheimer’s disease. Ageing Research Reviews.

[66] Han SD, Bondi MW (2008). Revision of the apolipoprotein E compensatory mechanism recruitment hypothesis. Alzheimers & Dementia.

[67] Flory JD, Manuck SB, Ferrell RE, Ryan CM, Muldoon MF (2000). Memory performance and the apolipoprotein E polymorphism in a community sample of middle-aged adults. American Journal of Medical Genetics.

[68] Greenwood PM, Lambert C, Sunderland T, Parasuraman R (2005). Effects of apolipoprotein E genotype on spatial attention, working memory, and their interaction in healthy, middle-aged adults: Results from the National Institute of Mental Health’s BIOCARD study. Neuropsychology.

[69] Greenwood PM, Sunderland T, Putnam K, Levy J, Parasuraman R (2005). Scaling of visuospatial attention undergoes differential longitudinal change as a function of APOE genotype prior to old age: Results from the NIMH BIOCARD study. Neuropsychology.

[70] Dik MG (2001). Memory complaints and APOE-epsilon 4 accelerate cognitive decline in cognitively normal elderly. Neurology.

[71] Cansino S (2015). fMRI subsequent source memory effects in young, middle-aged and old adults. Behavioural Brain Research.

[72] Kwon D (2016). Context Memory Decline in Middle Aged Adults is Related to Changes in Prefrontal Cortex Function. Cerebral Cortex.

[73] Salvato G, Patai EZ, McCloud T, Nobre AC (2016). Apolipoprotein epsilon 4 breaks the association between declarative long-term memory and memory-based orienting of spatial attention in middle-aged individuals. Cortex.

[74] Manelis A, Reder LM (2012). Procedural learning and associative memory mechanisms contribute to contextual cueing: Evidence from fMRI and eye-tracking. *Learning &*. Memory.

[75] Cohen MA, Horowitz TS, Wolfe JM (2009). Auditory recognition memory is inferior to visual recognition memory. Proceedings of the National Academy of Sciences of the United States of America.

[76] Kastner, S. Mechanisms of visual attention in the human cortex. *Journal of Cognitive Neuroscience*, 12–12 (2000). 10.1146/annurev.neuro.23.1.315 10845067

[77] Corbetta M, Shulman GL (2002). Control of goal-directed and stimulus-driven attention in the brain. Nature Reviews Neuroscience.

[78] Giesbrecht B, Woldorff MG, Song AW, Mangun GR (2003). Neural mechanisms of top-down control during spatial and feature attention. Neuroimage.

[79] Smith DV (2010). Spatial Attention Evokes Similar Activation Patterns for Visual and Auditory Stimuli. Journal of Cognitive Neuroscience.

[80] Harasty JA, Halliday GM, Kril JJ, Code C (1999). Specific temporoparietal gyral atrophy reflects the pattern of language dissolution in Alzheimer’s disease. Brain.

[81] Tuwaig M (2017). Deficit in Central Auditory Processing as a Biomarker of Pre-Clinical Alzheimer’s Disease. Journal of Alzheimers Disease.

[82] Izaks Gerbrand J., Gansevoort Ron T., van der Knaap Aafke M., Navis Gerjan, Dullaart Robin P. F., Slaets Joris P. J. (2011). The Association of APOE Genotype with Cognitive Function in Persons Aged 35 Years or Older. PLoS ONE.

[83] Rawle, M. J. *et al*. Apolipoprotein-E (Apoe) epsilon 4 and cognitive decline over the adult life course. *Translational**Psychiatry* **8**, 10.1038/s41398-017-0064-8 (2018). 10.1038/s41398-017-0064-8 PMC5802532 29317609

